# Supplementary material for: Exercise Affects Blood Glucose Levels and Tissue Chromium Distribution in High-Fat Diet-Fed C57BL6 Mice
Source: Molecules. 2020 Apr 3;25(7):1658. doi: 10.3390/molecules25071658 (PMC7180458; doi:10.3390/molecules25071658)
Supplement: Supplementary file 1 [file molecules-25-01658-s001.pdf]

Table S1. Effects of exercise on blood glucose, serum insulin levels, and chromium levels in organs and tissues from control- or exercise-trained mice in a SD.

Variable	SD	SD+EX
Body weight (g)	26.17 ± 0.41	25.98 ± 0.72
Food intake/mouse/day (g)	4.31 ± 0.02	4.28 ± 0.04
Blood glucose (mmol/L)	4.06 ± 0.14	4.03 ± 0.09
Serum insulin (pg/mL)	42.05 ± 0.87	41.63 ± 0.17
Blood (ppb) (x 10^2)	1.33 ± 0.08	1.18 ± 0.04
Bone (ppb) (x 10^2)	3.21 ± 0.04	3.05 ± 0.17
Muscle (ppb) (x 10^2)	1.21 ± 0.05	1.23 ± 0.13
Liver (ppb) (x 10^2)	1.48 ± 0.25	1.60 ± 0.20
Epididymal fat pads (ppb) (x 10^2)	1.23 ± 0.10	1.41 ± 0.12
Kidney (ppb) (x 10^2)	1.37 ± 0.14	1.39 ± 0.10

[1]Mice received exercise protocol or control on a standard diet (SD, diet 5008, 23.5% protein-enriched diet, 49.4% carbohydrates, 1.4 ppm Cr, metabolizable energy 3.3 kcal/gm, PMI Nutrition International Inc, MO, USA) for 12 weeks.

[2]Data are presented as means ± SEM. n = 8 for all groups. No statistical significance between the two groups.

Table S2. Chemical composition of a standard diet (Diet 5008) and high-fat diet (Diet 592Z).

Variable	LabDiet 5008	TestDiet 592Z
Protein (%)	23.5	20.4
Fat (%)	6.5	37.2
Fiber (%)	3.8	4.5
Carbohydrates (%)	49.4	21.5
Ash (%)	6.8	6.3
Lard (%)	-	35.5
Metabolizable energy (kcal/gm)	3.3	4.5
Chromium (ppm)	1.4	1.12
Calories provided by:		
Protein (%)	26.85	16.24
Fat (%)	16.71	66.63
Carbohydrates (%)	56.44	17.13
